# Engineering *Aspergillus niger* for galactaric acid production: elimination of galactaric acid catabolism by using RNA sequencing and CRISPR/Cas9

**DOI:** 10.1186/s12934-016-0613-5

**Published:** 2016-12-12

**Authors:** Joosu Kuivanen, Y.-M. Jasmin Wang, Peter Richard

**Affiliations:** VTT Technical Research Centre of Finland Ltd, P. O. Box 1000, FI-02044 Espoo, Finland

**Keywords:** *Aspergillus niger*, Metabolic engineering, CRISPR, Pectin, d-galacturonic acid, Galactaric acid, Mucic acid, Uronate dehydrogenase

## Abstract

**Background:**

*meso*-Galactaric acid is a dicarboxylic acid that can be produced by the oxidation of d-galacturonic acid, the main constituent of pectin. Mould strains can be engineered to perform this oxidation by expressing the bacterial enzyme uronate dehydrogenase. In addition, the endogenous pathway for d-galacturonic acid catabolism has to be inactivated. The filamentous fungus *Aspergillus niger* would be a suitable strain for galactaric acid production since it is efficient in pectin hydrolysis, however, it is catabolizing the resulting galactaric acid via an unknown catabolic pathway.

**Results:**

In this study, a transcriptomics approach was used to identify genes involved in galactaric acid catabolism. Several genes were deleted using CRISPR/Cas9 together with in vitro synthesized sgRNA. As a result, galactaric acid catabolism was disrupted. An engineered *A. niger* strain combining the disrupted galactaric and d-galacturonic acid catabolism with an expression of a heterologous uronate dehydrogenase produced galactaric acid from d-galacturonic acid. The resulting strain was also converting pectin-rich biomass to galactaric acid in a consolidated bioprocess.

**Conclusions:**

In the present study, we demonstrated the use of CRISPR/Cas9 mediated gene deletion technology in *A. niger* in an metabolic engineering application. As a result, a strain for the efficient production of galactaric acid from d-galacturonic acid was generated. The present study highlights the usefulness of CRISPR/Cas9 technology in the metabolic engineering of filamentous fungi.

**Electronic supplementary material:**

The online version of this article (doi:10.1186/s12934-016-0613-5) contains supplementary material, which is available to authorized users.

## Background

*meso*-Galactaric acid, also known as mucic acid, is a hexaric acid i.e. a sugar acid with two terminal carboxyl groups. It is an attractive platform chemical that can be produced from biomass. It is used as such, e.g. in skincare products, or, more importantly, can be chemically converted to monomers for polymer production. It can be reduced to adipic acid [1] which is a precursor for Nylon or converted to 2,5-furandicarboxylic acid (FDCA) [2, 3]. FDCA is considered as promising renewable that has the potential to replace the fossil-based terephthalic acid. Terephtalic acid is used to produce polyethylene terephthalate, PET, that is used for bottles for carbonated soft drinks. The FDCA based polymer would have similar or even superior properties [4]. An attractive raw material for galactaric acid production is d-galacturonic acid (d-galUA). d-GalUA is the most abundant monomer in pectin which is a component of plant primary cell wall and especially abundant in non-woody plant biomass such as in fruit peels. Several pectin rich waste biomass streams, such as residues from citrus fruit and sugar beet processing are available and currently poorly utilized. Galactaric acid can be produced from d-galUA via chemical oxidation [5], however an attractive alternative is the engineering of a microorganism to perform this reaction.

**Fig. 1:**
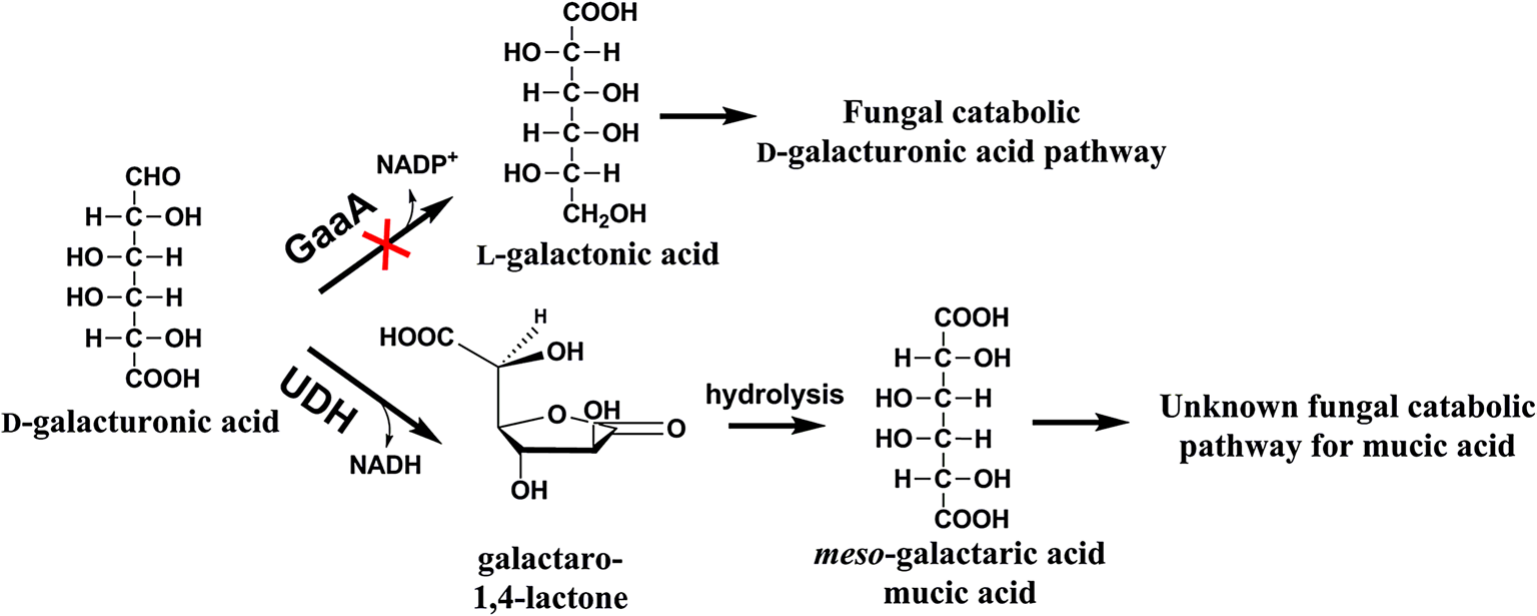
The first enzyme, GaaA, in the fungal catabolic d-galacturonic acid pathway in *A. niger* and a heterologous uronate dehydrogenase (UDH) for galactaric acid production

In the bacterial pathway for oxidative d-galUA catabolism, galactaric acid is a metabolite. In the pathway, an uronate dehydrogenase (UDH) oxidizes d-galUA resulting in formation of galactaro-1,4-lactone (Fig. 1) [6]. After the spontaneous hydrolysis of the lactone, galactaric acid is formed. The end product of this pathway is α-ketoglutarate, an intermediate of TCA cycle, [7, 8]. The *udh* gene has been used to engineer different microbes for the oxidation of d-galUA such as the yeast *Saccharomyces cerevisiae* [9], the bacterium *Escherichia coli* [10] and the moulds *Trichoderma reesei* and *Aspergillus niger* [11]. The use of moulds has the advantage that these organisms are often efficient producers of enzymes to hydrolyse the biomass, which would facilitate a consolidated process in which pectin-rich biomass could be hydrolysed and converted to galactaric acid in a single process. Moulds are naturally consuming d-galUA using a reductive pathway [12]. The first reaction is the reduction of d-galUA by the action of a reductase [13]. This gene has to be deleted in combination with the expression of an UDH to produce galactaric acid (Fig. 1). In *T. reesei* this was efficient; the deletion of the d-galUA reductase in combination with the expression of a UDH resulted in a strain that was almost on an equimolar basis converting d-galUA to galactaric acid. In *A. niger*, this was less efficient and a strain with a deletion in the d-galUA reductase expressing a UDH showed growth on d-galUA [11]. This suggested that *A. niger* can use galactaric acid as a carbon source. *A. niger* would be more suitable for conversion of pectin-rich biomass since this mould is producing more efficiently pectinases as compared to *T. reesei*, but it would require that galactaric acid catabolism is disabled. However it is not known how galactaric acid is catabolised in fungi.

In the present study, we aimed to disrupt the catabolism of galactaric acid in *A. niger*. We used RNA sequencing to identify genes involved in galactaric acid catabolism. Several genes were deleted using CRISPR/Cas9 together with in vitro synthesized single chimeric guide RNA (sgRNA). As a result, the galactaric acid catabolism was disrupted in *A. niger* and a strain capable of galactaric acid production from d-galUA was generated.

## Results

### RNA sequencing

**Fig. 2:**
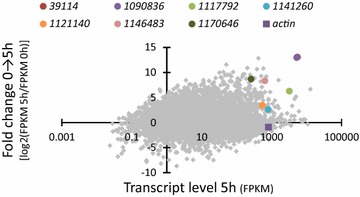
RNA sequencing of *A. niger* ATCC 1015 at 0 h and 5 h after the shift to galactaric acid. Fold change in transcript levels between 0 and 5 h on y-axis and transcript levels after 5 h on x-axis. Transcript levels are presented as fragments per kilobase of exon per million fragments mapped (FPKM). The protein ID numbers refer the numbers from the Join Genome Institute, MycoCosm, *A. niger* ATCC 1015 v.4.0 database

*Aspergillus niger* wild type mycelium was cultivated on galactaric acid and the utilization was monitored using HPLC (data not shown). It was indeed confirmed that *A. niger* is capable of catabolizing galactaric acid and about 2 gl^−1^ of the initial 10 gl^−1^ galactaric acid was consumed after 18 h. In addition, we observed that oxalic acid was produced during the cultivation. In order to discover the genes encoding enzymes involved in galactaric acid catabolism, samples for RNA sequencing were collected after 0, 5 and 18 h. The results from the RNA sequencing are presented in Fig. 2 and the detailed data in Additional file 1: Table S1. Fig. 2 describes the induction of transcript levels between 0 and 5 h (Y-axis) and the absolute transcript levels at 5 h (X-axis). We selected seven genes that were induced on galactaric acid (Fig. 2, values on Y-axis clearly above 1), had absolute transcript levels around similar or higher than that of actin at 5 h (Fig. 2, X-axis) and are predicted to code for a protein that could be involved in carbohydrate metabolism, such as oxidoreductases (Table 1; Additional file 1: Table S1). In addition, genes putatively encoding transport proteins (such as IDs 1084943, 1086238, 1088440, 1094471 and 1090078) and a putative transcriptional regulatory protein (ID 1125622) were among the most induced genes between 0 and 5 h (Additional file 1: Table S1). However, with these genes the absolute transcript levels at 5 and 18 h were relatively low when compared to the seven selected genes.

**Table 1:** Genes selected for deletion based on RNA sequencing of *A. niger* wild type strain cultivated on galactaric acid

Protein ID	Galactaric acid			InterPro/KOG prediction
	0 h	5 h	18 h	
39114	1	5497	4219	AMP-dependent synthetase and ligase, α-aminoadipate-semialdehyde dehydrogenase
1090836	1	5239	6627	Aldo/keto reductase
1117792	42	3239	1050	Alcohol dehydrogenase, zinc-binding
1141260	132	805	747	Short-chain dehydrogenase/reductase
1121140	51	545	1162	FAD-dependent oxidoreductase
1146483	2	641	1661	Mandelate racemase/muconate lactonizing enzyme
1170646	1	260	164	d-isomer specific 2-hydroxyacid dehydrogenase

Transcript levels are presented as fragments per kilobase of exon per million fragments mapped (FPKM). The protein ID numbers refer the numbers from the Join Genome Institute, MycoCosm, *A. niger* ATCC 1015 v.4.0 database

### CRISPR/Cas9 mediated gene deletions

The selected seven genes (Table 1), putatively encoding metabolic enzymes, were deleted from the uracil auxotrophic strain *A. niger ∆pyrG*. We used deletion cassettes containing homologous flanking regions (1.5 kb) for the target gene and *pyrG* as selectable marker. One of the genes (ID 39114) was deleted by using only the deletion cassette and uracil-deficient medium for selection. For rest of the genes, we used CRISPR/Cas9 technology implemented through the *AMA* plasmid pFC-332 [14] expressing Cas9 together with the selectable marker *hyg* for hygromycin. Instead of expressing the sgRNA from the plasmid, we used two in vitro synthetized sgRNAs for each gene, which were delivered together with the Cas9 plasmid and deletion cassette in the transformation. The sgRNAs were targeted to different parts of the gene in order to release the gene. The deletion cassette, after integration by homologous recombination, would then introduce the *pyrG* at the location of the gene. Uracil-deficient medium supplemented with hygromycin was used in the CRISPR/Cas9 transformations generating selection pressure for the cassette and for the Cas9 plasmid. For four of the target genes both deletion methods were used. The frequency of correct gene deletions improved dramatically when Cas9 and in vitro synthesized sgRNAs were used (Table 2).

**Table 2:** Frequency of the correct gene deletion with and without CRISPR/Cas9

	Protein ID	Screened	Correct	Frequency (%)
No CRISPR/Cas9	39114	30	2	6.7
	1090836	30	1	3.3
	1117792	30	13	43.3
	1141260	30	0	0.0
	1121140	60	1	1.7
CRISPR/Cas9 + in vitro sgRNA	1090836	40	11	27.5
	1117792	8	8	100
	1141260	8	8	100
	1121140	8	3	37.5
	1146483	8	7	87.5
	1170646	8	5	62.5

### Disruption of galactaric acid catabolism

**Fig. 3:**
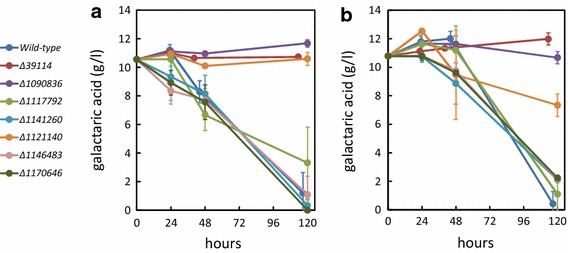
Consumption of galactaric acid in liquid cultivations on 24-well plates in **a** minimal medium with d-xylose and **b** YP-medium. Data represent mean ± standard deviation from four replicates

The consumption of galactaric acid in minimal medium as observed in the cultivations for the RNA sequencing was slow and not complete (data not shown). To accelerate the cultivations of the mutant strains we used a medium that contained also additional carbon sources together with galactaric acid (Fig. 3). We tested the mutant strains in liquid cultivation in minimal medium containing galactaric acid and d-xylose (Fig. 3a) and galactaric acid in YP-medium (Fig. 3b). With three of the mutant strains—*∆1141260*, *∆1146483* and *∆1170646*—in the d-xylose medium, galactaric acid consumption was observed during the first 24 h while the wild type and *∆1117792* started to consume galactaric acid later. Three of the mutants—*∆39114*, *∆1090836* and *∆1121140*—did not consume galactaric acid in these conditions. With the YP-medium, a short lag phase was observed before galactaric acid consumption started by the wild type and five of the mutant strains. Similar to the cultivations on d-xylose medium, early galactaric acid consumption by the three mutants was observed also on YP-medium. In the end, both conditions resulted in similar observation; strains *∆39114*, *∆1090836* and *∆1121140* had completely disrupted or reduced catabolism of galactaric acid. In the case of *∆39114*, the catabolism was completely blocked in both conditions while *∆1090836* and *∆1121140* showed some, however reduced galactaric acid consumption on YP-medium.

### Engineering *A. niger* for galactaric acid production

In order to engineer *A. niger* for galactaric acid production, the gene with the ID 39114 was deleted from the *A. niger* strain *∆gaaA*-*udh*. The strain *∆gaaA*-*udh* has a disrupted pathway for d-galUA catabolism (deletion of *gaaA*); however, introduction of a UDH restored the catabolism of d-galUA and the strain produced only small amounts of galactaric acid [11]. The strain was uracil prototroph (+*pyrG*) but we decided to use the same deletion cassette containing *pyrG* selectable marker for 39114 which was used in the initial gene deletions from *∆pyrG* strain. This time we combined the deletion cassette with the Cas9 plasmid and in vitro synthesized sgRNA. Consequently, the selection pressure was only for the Cas9 plasmid but not for the donor DNA. Nevertheless, 2 out of 24 screened colonies (8.3%) revealed the correct gene deletion.

**Fig. 4:**
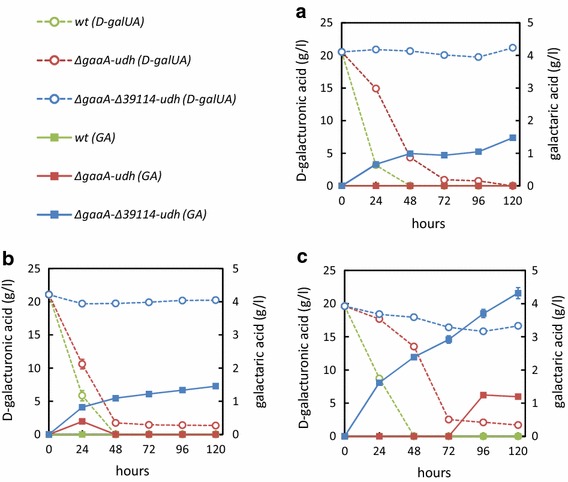
Production of galactaric acid (GA, *squares*) from d-galacturonic acid (d-galUA, *open circles*) in shake flask cultivations on **a** minimal medium with d-galacturonic acid, **b** minimal medium with d-xylose and d-galacturonic acid and **c** YP-medium with d-galacturonic acid. The strains are wild type (*green symbols*), *∆gaaA*-*udh* (*red symbols*) and *∆gaaA*-*∆39114*-*udh* (*blue symbols*). Data represent mean ± standard deviation from three replicates. If *error bars* are not visible they are smaller than the symbol

Next the resulting strain *∆gaaA*-*∆39114*-*udh* was tested for galactaric acid production in shake flask cultivations on d-galUA (Fig. 4). Galactaric acid concentrations of around 1.5 gl^−1^ were observed in the minimal medium without (Fig. 4a) and with (Fig. 4b) co-substrate (d-xylose) by the *∆gaaA*-*∆39114*-*udh* strain while the *∆gaaA*-*udh* did not accumulate galactaric acid. On rich YP-medium, the production increased and reached values above 4 gl^−1^ by the *∆gaaA*-*∆39114*-*udh* (Fig. 4c). The strain *∆gaaA*-*udh* started to produce galactaric acid after 96 h; however, values remained about four-fold lower when compared to the *∆gaaA*-*∆39114*-*udh* strain (Fig. 4c). In terms of product yields, the *∆gaaA*-*∆39114*-*udh* was superior; approximately all the consumed d-galUA was converted to galactaric acid. With the *∆gaaA*-*udh* strain, only about 7% of consumed d-galUA was converted to galactaric acid.

**Fig. 5:**
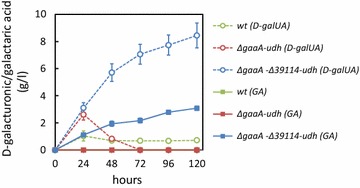
Consolidated bioprocess from orange processing waste by the wild type strain (*green symbols*) and *∆gaaA*-*∆39114*-*udh* (*blue symbols*). Concentrations of d-galacturonic acid (d-galUA, *open circles*) and galactaric acid (GA, *squares*) are presented. Data represent mean ± standard deviation from three replicates. If *error bars* are not visible they are smaller than the symbol

We also tested whether the strain could be used in a consolidated process for the production of galactaric acid directly from pectin-rich biomass. Processing waste from an orange juice industry was used as substrate in submerged cultivations (Fig. 5). As a result, 3.1 gl^−1^ galactaric acid was produced from 37.4 gl^−1^ (dry mass) orange processing waste by the *∆gaaA*-*∆39114*-*udh*. The content of d-galUA in the waste is about 27% [15] resulting in a maximum theoretical galactaric acid concentration of around 10 gl^−1^ that can be achieved. In addition to galactaric acid, 8.4 gl^−1^ free d-galUA was observed in the cultivations after 120 h. The sum of observed galactaric acid and d-galUA corresponds approximately to the total d-galUA content in the orange processing waste. In contrast, the wild type strain and *∆gaaA*-*udh* consumed most of the d-galUA released from the substrate and only low concentrations were observed during the cultivations. In addition, no galactaric acid was observed with the *∆gaaA*-*udh*. To sum up, the process for consolidated galactaric acid production resulted in the product titer of about 30% of theoretical maximum by the *∆gaaA*-*∆39114*-*udh*.

## Discussion

The mould *A. niger* is a widely used organism for the commercial production of enzymes and organic acids. It has also the natural capacity to degrade different biomass polymers. Pectin is one such a polymer abundantly available in some agro-industrial waste streams. Galactaric acid is an attractive dicarboxylic acid that can be produced by biochemical oxidation of d-galUA, the main constituent of pectin. *Aspergillus niger* can be engineered for the oxidation, however, it catabolizes the resulting galactaric acid. In this study, we used the advanced technologies—RNA sequencing and CRISPR/Cas9—to overcome this problem. As a result, we generated an engineered strain with disrupted d-galUA and galactaric acid catabolism together with heterologously expressed UDH producing galactaric acid from d-galUA.

The RNA sequencing of *A. niger* cultivated on galactaric acid as sole carbon source reveled several specifically induced genes putatively encoding enzymes and transport proteins that may have a function in galactaric acid catabolism. Also many other genes, not relevant for carbohydrate metabolism, had altered transcription levels after the shift to galactaric acid medium. This may be partially due to the different composition of the pre-cultivation medium (yeast extract peptone) and galactaric acid medium (minimal medium). Although not studied in detailed in aspergilli, the genes of the most central carbon metabolic pathways, such as glycolytic genes, are considered to be constitutively active at least in the model organism *Saccharomyces cerevisiae* [16]. In contrast, metabolic pathways for less abundant carbon sources, such as the catabolic pathways for d-galUA [17] and d-glucuronic acid [18] in *A. niger*, tend to be specifically activated in the presence of a particular carbon source. This seems to be the case also with the catabolic galactaric acid pathway in *A. niger*. The genes putatively encoding a glucose-6-phosphate isomerase (ID 1145755)—a glycolytic enzyme—and a glucose-6-phospate dehydrogenase (ID 1145051)—the first enzyme in the pentose phosphate pathway—were both constitutively transcribed across all the three time points (Additional file 1: Table S1). In contrast, the genes 39114, 1090836 and 1121140 involved in galactaric acid catabolism had almost no transcription at 0 h whereas the transcription was highly induced after 5 and 18 h on galactaric acid. As such, it is surprising that *A. niger* has a metabolic pathway for galactaric acid catabolism since most likely galactaric acid is only rarely available in the natural environment.

We used CRISPR/Cas9 technology to accelerate the generation of mutant strains with disrupted galactaric acid catabolism. The non-homologous end joining (NHEJ) pathway is the predominant mechanism for DNA repair in *A. niger*. Thus the frequency of homologous recombination is typically low in transformations. Previously, CRISPR/Cas9 mediated gene deletions were described in several *Aspergillus* species by using the *AMA* plasmid expressing both Cas9 protein and sgRNA [14]. Due to the poor availability of characterized RNA polymerase (RNA pol) III promoters, sgRNA was expressed under a RNA pol II promoter which requires the use of additional ribozyme structures to release a functional sgRNA. In *A. niger*, transformation of the plasmid without donor DNA resulted in successful gene disruptions via short deletions by the NHEJ mediated repair. In the present study, we used in vitro synthesized sgRNAs. This approach overcomes the time consuming DNA construction for sgRNA expression cassettes and prevents other possible problems in the sgRNA expression. In addition, the simultaneous use of multiple sgRNAs is easy. This approach has been described earlier in the filamentous fungi *Trichoderma reesei* [19], *Penicillium chrysogenum* [20] and *Aspergillus fumigatus* [21] but not in *Aspergillus niger*. We also decided to use deletion cassettes as donor DNA that contained *pyrG*. This approach allowed double selection resulting in high frequencies of correct deletions. The use of donor DNA allows also easier screening of the genotypes by colony PCR which would not necessary detect short deletions in the genome resulting from NHEJ pathway repair without donor DNA.

Seven different genes with induced transcription on galactaric acid were selected for further investigation. Three of the gene deletion mutants showed earlier galactaric acid consumption when compared to the wild type. We hypothesize that this may be due to slight differences in growth phases during the cultivations, for example, arising from variations in orotidine 5′-phosphate carboxylase activities (*pyrG*) used as selection marker in the gene deletion cassettes. Three gene deletion mutants—Δ1121140, Δ1090836, Δ39114—with disrupted galactaric acid catabolism were identified in the initial screen. Some degree of galactaric acid consumption was observed by the mutant strains Δ1121140 and Δ1090836 on YP-medium. This can be derived from earlier pathway reactions converting galactaric acid to pathway intermediates if the protein products of 1121140 and 1090836 are acting later in the pathway or due to compensating enzyme activities by other proteins present when the mutant strain is cultivated on YP-medium. The strongest phenotype was observed when the gene with the ID 39114 was deleted. The protein product of the gene 39114 has a predicted function of α-aminoadipate-semialdehyde dehydrogenase (EC 1.2.1.31) also known as α-aminoadipate reductase (AAR) [22]. The AAR catalyzes the reduction of a carboxyl group in α-aminoadipate resulting in formation of α-aminoadipate-semialdehyde in the fungal biosynthetic lysine pathway [22]. Similar to α-aminoadipate, galactaric acid is also a dicarboxylic acid and may be reduced by the action of 39114. In order to investigate the enzymatic activity, we expressed the open reading frame of 39114 in yeast and tried to assay the activity from the resulting cell crude extract (description and data not shown). We tested the oxidoreductase activity towards galactaric acid with NAD^+^, NADH, NADP^+^ and NADPH, however, we were unable to show the activity. It is possible that 39114 have the activity later in the galactaric acid pathway or the expression in yeast resulted in an inactive protein product. The protein products of the genes with the IDs 1090836 and 1121140 have predicted functions of aldo/keto reductase and FAD-dependent oxidoreductase, respectively. Enzymatic activities of these proteins also remain unknown.

Deletion of the gene 39114 from the strain *∆gaaA*-*udh* resulted in a strain that produces galactaric acid from d-galUA at an equimolar ratio. In the cultivations on minimal and YP-medium, all the consumed d-galUA was converted to galactaric acid by the strain *∆gaaA*-*∆39114*-*udh*. However, a big fraction of the available d-galUA was not converted in the process. Although the oxidation of d-galUA by the action of UDH generates energy in the form of NADH it doesn’t seem to be sufficient to maintain the process. Thus, an optimal co-substrate feed is required in order to achieve the complete conversion of d-galUA. We did not optimize the process, however it is expected that with a careful process design, higher galactaric acid yields can be achieved. In the case of consolidated bioprocess from citrus processing waste, more additional carbon sources are available. In fact, the process from orange peel waste to galactaric acid resulted in a higher yield than the process from pure d-galUA with d-xylose or YP as co-substrate; the concentrations of galactaric acid and residual d-galUA in the consolidated process were 3.1 and 8.4 gl^−1^, respectively, while the same concentrations in the cultivation on YP-medium were 4.3 and 16.7 gl^−1^, respectively.

## Conclusions

In the present study, we demonstrated the use of CRISPR/Cas9 mediated gene deletion technology in *A. niger* in an metabolic engineering application. The use of in vitro synthesized sgRNA in CRISPR/Cas9 technology was reported for the first time in *A. niger*. As a result, the catabolism of galactaric acid, an industrially useful platform chemical, was disrupted and a strain for the efficient production of galactaric acid from d-galUA was generated. The consolidated process from pectin-rich biomass for the production of galactaric acid was also demonstrated. The present study highlights the usefulness of CRISPR/Cas9 technology in the metabolic engineering of filamentous fungi.

## Methods

### Strains

The *Aspergillus niger* strain ATCC 1015 (CBS 113.46) was used as a wild type. The *A. niger ∆pyrG* strain (deleted orotidine-5′-phosphate decarboxylase) and the platform strain for galactaric acid production *∆gaaA*-*udh* (deleted d-galUA reductase and *Agrobacterium tumefaciens* uronate dehydrogenase, UDH, introduced through random genomic integrations) were described previously [11]. All the plasmids were produced in *Escherichia coli* TOP10 cells. The *Saccharomyces cerevisiae* strain ATCC 90845 was used in the homologous recombination for the construction of deletion cassettes.

### Media and culture conditions

Luria Broth culture medium supplemented with 100 µg ml^−1^ of ampicillin and culture conditions of 37 °C and 250 rpm were used for *E. coli* cultures. YP-medium (10 g yeast extract l^−1^, and 20 g peptone l^−1^) supplemented with 20 g d-glucose l^−1^ was used for yeast pre-cultures. After the transformations in yeast, SCD-URA (uracil deficient synthetic complete media supplemented with 20 g d-glucose l^−1^) plates were used for uracil auxotrophic selection. All the yeast cultivations were carried out at 30 °C and the liquid cultivations at 250 rpm. *Aspergillus niger* spores were generated on potato-dextrose plates and ~10^8^ spores were inoculated to 50 ml of YP medium (10 g yeast extract l^−1^, 20 g peptone l^−1^) containing 30 g gelatin l^−1^ for pre-cultivations. Mycelia were pre-grown in 250-ml Erlenmeyer flasks by incubating overnight at 28 °C, 200 rpm and harvested by vacuum filtration, rinsed with sterile water and weighted. In *A. niger* transformations, *A. nidulans* defined minimal medium [23] plates supplemented with 1.2 M d-sorbitol and 20 g agar l^−1^ and, in the case of CRISPR/Cas9 transformations, 400 µg/ml hygromycin were used. The minimal medium used in the phenotypic characterization in liquid cultivations contained 10 g galactaric acid l^−1^ with or without 5 g d-xylose l^−1^ and the pH was adjusted to 7.0. These cultivations were inoculated with 10 gl^−1^ (wet) of pre-grown mycelia. Alternatively, YP-medium supplemented with 10 g galactaric acid l^−1^ was used. Similar minimal or YP-medium supplemented with 20 g d-galUA l^−1^ pH 5 was used in the cultivations for galactaric acid production and were inoculated with 10 gl^−1^ (wet) of pre-grown mycelia. For the consolidated process, the minimal medium was supplemented with 40 gl^−1^ of orange processing waste as described earlier [24].

### Transcriptional analysis

*Aspergillus niger* wild type strain ATCC 1015 was cultivated in the minimal medium supplemented with 10 g galactaric acid l^−1^. Samples of 2 ml were collected and the mycelium was harvested by vacuum filtration. The filtered mycelium was frozen with liquid nitrogen and stored at −80 °C. Total RNA was extracted using the RNeasy Plant Mini Kit (Qiagen). RNA library preparation and sequencing was carried out by GATC (Constance, Germany) using the InView™ Transcriptome Explore package. The raw data was processed as described earlier [18]. The protein ID numbers refer the numbers from the Join Genome Institute, MycoCosm, *A. niger* ATCC 1015 v.4.0 database (http://genome.jgi.doe.gov/Aspni7/Aspni7.home.html) [25].

### Gene deletions

For the deletion of the genes identified in the RNA sequencing, deletion cassettes containing homologous 5′ and 3′ flanks (~1.5 kb) for targeted integration and the selectable marker *pyrG* (*A. niger*) were constructed. The 5′ and 3′ flanks were amplified by PCR (KAPA HiFi DNA polymerase, Kapa Biosystems) with the primers described in Additional file 2: Table S2. The amplified flanks and *pyrG* were joined with the *EcoRI*/*BamHI* (Thermo) digested pRS426 plasmid using yeast homologous recombination. The resulting plasmids were amplified in *E. coli* and the cassettes linearized with *NotI* (Thermo). The linearized cassettes (10 µg) were transformed to *A. niger ∆pyrG* strain with or without the Cas9 plasmid pFC-332 (1 µg) [14] and two suitable in vitro synthesized sgRNAs (10 µg) (GeneArt™ Precision Synthesis Kit) as described in Additional file 2: Table S2. For the generation of a galactaric acid producing strain, the gene with the ID 39114 was deleted from the strain *∆gaaA*-*udh*. All the *A. niger* transformations were carried out using the protoplast transformation method. Correct integration of the transformed cassette into the genome and disappearance of the open reading frame of the target gene was confirmed with colony PCR using Phire direct PCR kit (Thermo) and the primers listed in Additional file 2: Table S2.

### Chemical analyses

Samples were removed from liquid cultivations at intervals and mycelium was separated from the supernatant by filtration. The concentration of galactaric acid and d-galUA was determined by HPLC using a Fast Acid Analysis Column (100 mm × 7.8 mm, BioRad Laboratories, Hercules, CA) linked to an Aminex HPX-87H organic acid analysis column (300 × 7.8 mm, BioRad Laboratories) with 5.0 mM H_2_SO_4_ as eluent and a flow rate of 0.5 ml min^−1^. The column was maintained at 55 °C. Peaks were detected using a Waters 2489 UV/Visible dual wavelength UV (210 nm) detector.

## Additional files

**Additional file 1: Table S1.** RNA sequencing data of *A. niger* wild type strain pre-cultivated on YP-medium (0 hour) and cultivated to galactaric acid medium (5 and 18 hours). Transcript levels are presented as fragments per kilobase of exon per million fragments mapped (FPKM).

**Additional file 2: Table S2.** DNA primer and sgRNA sequences used in the study.

## Abbreviations

d-galUA: d-galacturonic acid
UDH: uronate dehydrogenase
*A. niger*: *Aspergillus niger*
*T. reesei*: *Trichoderma reesei*
CRISPR: clustered regularly interspaced short palindromic repeats
sgRNA: single chimeric guide RNA
FDCA: 2,5-furandicarboxylic acid
PET: polyethylene terephthalate
NHEJ: non-homologous end joining
AAR: α-aminoadipate reductase

## References

[1] Li X, Wu D, Lu T, Yi G, Su H, Zhang Y (2014). Highly efficient chemical process to convert mucic acid into adipic acid and DFT studies of the mechanism of the rhenium-catalyzed deoxydehydration. Angewandte Chemie.

[2] Lewkowski J (2001). Convenient synthesis of furan-2,5-dicarboxylic acid and its derivatives. Polish J. Chem.

[3] Taguchi Y, Oishi A, Iida H (2008). One-step synthesis of dibutyl furandicarboxylates from galactaric acid. Chem Lett.

[4] de Jong E, Dam M, Sipos L, Gruter G-J, Smith P, Gross R (2012). Furandicarboxylic acid (FDCA), a versatile building block for a very interesting class of polyesters. Biobased monomers, polym mater. ACS symposium series.

[5] Rautiainen S, Lehtinen P, Chen J, Vehkamäki M, Niemelä K, Leskelä M (2015). Selective oxidation of uronic acids into aldaric acids over gold catalyst. RSC Adv..

[6] Boer H, Maaheimo H, Koivula A, Penttilä M, Richard P (2010). Identification in *Agrobacterium tumefaciens* of the d-galacturonic acid dehydrogenase gene. Appl Microbiol Biotechnol.

[7] Dagley S, Trudgill PW (1965). The metabolism of galactarate, d-glucarate and various pentoses by species of *Pseudomonas*. Biochem J.

[8] Chang YF, Feingold DS (1970). d-Glucaric acid and galactaric acid catabolism by *Agrobacterium tumefaciens*. J Bacteriol.

[9] Benz JP, Protzko RJ, Andrich JM, Bauer S, Dueber JE, Somerville CR (2014). Identification and characterization of a galacturonic acid transporter from *Neurospora crassa* and its application for *Saccharomyces cerevisiae* fermentation processes. Biotechnol Biofuels.

[10] Zhang H, Li X, Su X, Ang L, Zhang Y, Zhao H (2016). Production of adipic acid from sugar beet residue by combined biological and chemical catalysis. Chem Cat Chem..

[11] Mojzita D, Wiebe M, Hilditch S, Boer H, Penttila M, Richard P (2010). Metabolic engineering of fungal strains for conversion of d-galacturonate to meso-galactarate. Appl Environ Microbiol.

[12] Hilditch S, Berghäll S, Kalkkinen N, Penttilä M, Richard P (2007). The missing link in the fungal d-galacturonate pathway: identification of the l-threo-3-deoxy-hexulosonate aldolase. J Biol Chem.

[13] Kuorelahti S, Kalkkinen N, Penttilä M, Londesborough J, Richard P (2005). Identification in the mold *Hypocrea jecorina* of the first fungal d-galacturonic acid reductase. Biochemistry.

[14] Nodvig CS, Nielsen JB, Kogle ME, Mortensen UH (2015). A CRISPR-Cas9 system for genetic engineering of filamentous fungi. PLoS ONE.

[15] Kuivanen J, Dantas H, Mojzita D, Mallmann E, Biz A, Krieger N (2014). Conversion of orange peel to l-galactonic acid in a consolidated process using engineered strains of *Aspergillus niger*. AMB Express..

[16] Hackett SR, Zanotelli VRT, Xu W, Goya J, Park JO, Perlman DH (2016). Systems-level analysis of mechanisms regulating yeast metabolic flux. Science..

[17] Martens-Uzunova ES, Schaap PJ (2008). An evolutionary conserved d-galacturonic acid metabolic pathway operates across filamentous fungi capable of pectin degradation. Fungal Genet Biol.

[18] Kuivanen J, Sugai-Guérios MH, Arvas M, Richard P (2016). A novel pathway for fungal d-glucuronate catabolism contains an l-idonate forming 2-keto-l-gulonate reductase. Sci Rep..

[19] Liu R, Chen L, Jiang Y, Zhou Z, Zou G (2015). Efficient genome editing in filamentous fungus *Trichoderma reesei* using the CRISPR/Cas9 system. Cell Discov..

[20] Pohl C, Kiel JAK, Driessen AJM, Bovenberg RAL, Nygård Y (2016). CRISPR/Cas9 based genome editing of *Penicillium chrysogenum*. ACS Synth Biol.

[21] Zhang C, Meng X, Wei X, Lu L (2016). Highly efficient CRISPR mutagenesis by microhomology-mediated end joining in *Aspergillus fumigatus*. Fungal Genet Biol.

[22] Napora-Wijata K, Strohmeier GA, Winkler M (2014). Biocatalytic reduction of carboxylic acids. Biotechnol J.

[23] Barratt R, Johnson G, Ogata W (1965). Wild-type and mutant stocks of *Aspergillus nidulans*. Genetics.

[24] Kuivanen J, Penttilä M, Richard P (2015). Metabolic engineering of the fungal d-galacturonate pathway for l-ascorbic acid production. Microb Cell Fact.

[25] Andersen MR, Salazar MP, Schaap PJ, Van De Vondervoort PJI, Culley D, Thykaer J (2011). Comparative genomics of citric-acid-producing *Aspergillus niger* ATCC 1015 versus enzyme-producing CBS 513.88. Genome Res.

